# Can serum biomarkers predict the outcome of systemic immunosuppressive therapy in adult atopic dermatitis patients?

**DOI:** 10.1002/ski2.77

**Published:** 2022-01-07

**Authors:** G. Hurault, E. Roekevisch, M. E. Schram, K. Szegedi, S. Kezic, M. A. Middelkamp‐Hup, P. I. Spuls, R. J. Tanaka

**Affiliations:** ^1^ Department of Bioengineering Imperial College London London UK; ^2^ Department of Dermatology, Amsterdam Public health, Infection and Immunity Amsterdam UMC, Location AMC University of Amsterdam Amsterdam The Netherlands

## Abstract

**Background:**

Atopic dermatitis (AD or eczema) is a most common chronic skin disease. Designing personalised treatment strategies for AD based on patient stratification is of high clinical relevance, given a considerable variation in the clinical phenotype and responses to treatments among patients. It has been hypothesised that the measurement of biomarkers could help predict therapeutic responses for individual patients.

**Objective:**

We aim to assess whether serum biomarkers can predict the outcome of systemic immunosuppressive therapy in adult AD patients.

**Methods:**

We developed a statistical machine learning model using the data of an already published longitudinal study of 42 patients who received azathioprine or methotrexate for over 24 weeks. The data contained 26 serum cytokines and chemokines measured before the therapy. The model described the dynamic evolution of the latent disease severity and measurement errors to predict AD severity scores (Eczema Area and Severity Index, (o)SCORing of AD and Patient Oriented Eczema Measure) two‐weeks ahead. We conducted feature selection to identify the most important biomarkers for the prediction of AD severity scores.

**Results:**

We validated our model in a forward chaining setting and confirmed that it outperformed standard time‐series forecasting models. Adding biomarkers did not improve predictive performance.

**Conclusions:**

In this study, biomarkers had a negligible and non‐significant effect for predicting the future AD severity scores and the outcome of the systemic therapy.

## Graphical abstract

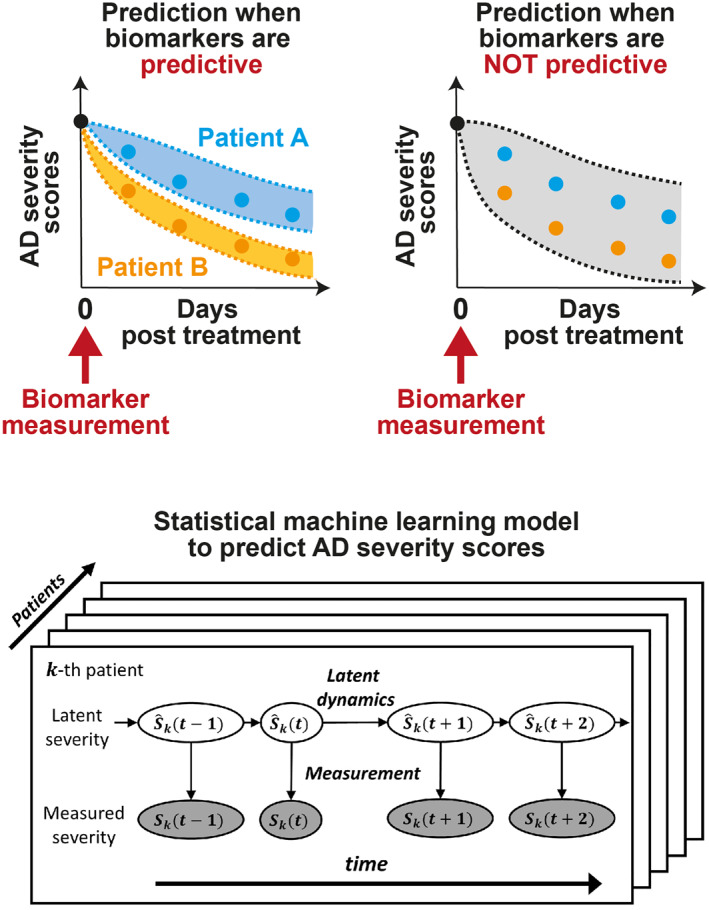

1

**What's already known about this topic?**

- Biomarker measurements could help predict therapeutic responses for atopic dermatitis (AD) and be used as a tool to stratify patients.
- Several studies aimed to explore ‘predictive’ biomarkers for AD treatments but did not investigate whether the biomarkers can predict treatment outcomes. Instead, they investigated how much the biomarkers were associated with treatment outcomes.
- An association does not imply prediction since associations often do not generalise to unseen data.

**What does this study add?**

- Serum biomarkers might not be as useful as expected for patient stratification for systemic immunosuppressive therapy for AD.
- A statistical machine learning approach can be used to analyse data from previous clinical trials and to design better and more informative future clinical trials.
- The repeated measurements of severity scores, even for a small number of patients, allow us to capture the dynamic nature of the AD severity scores and to investigate the consistent effects of biomarkers and treatments on AD severity scores within each patient.

## 2 INTRODUCTION

Atopic dermatitis is a chronic skin disease with a considerable variation in the clinical phenotype and responses to treatments among patients.^1^ Current treatments aim to manage AD symptoms, such as inflammatory flares and dry and itchy skin, mainly by topical application of emollients and corticosteroids. But systemic therapy using traditional immunosuppressants is needed for patients with moderate‐to‐severe AD that do not respond to topical therapy. It is desirable to identify patients who are likely to respond to a systemic immunosuppressive therapy, as the decision to initiate such therapy can be difficult given its known risks.^2^

It has been hypothesised that biomarker measurements could help predict therapeutic responses and be used as a tool to stratify patients.^3^ Previous studies on AD biomarkers have mainly focused on severity biomarkers, that is, biomarkers that could be used as surrogates for AD severity: thymus and activation‐regulated chemokine was suggested to be the single best biomarker to assess disease severity^4^ and panels of biomarkers were proposed as ‘objective’ substitutes for Eczema Area and Severity Index (EASI)^5^ and SCORing of AD (SCORAD).^6^ However, ‘severity’ biomarkers are different from ‘predictive’ biomarkers that are expected to be predictive of future outcomes.

Some previous studies aimed to explore ‘predictive’ biomarkers for several AD treatments. In Roekevisch et al. (2020),^7^ predictive biomarkers for systemic immunosuppressants (methotrexate or azathioprine) were sought by investigating whether baseline levels of some cytokines/chemokines are statistically different between responders (who achieved >50% reduction in SCORAD) and non‐responders of the therapy. In Kiiski et al. (2015),^8^ a high level of serum total IgE was found to be associated with poor response to the maintenance treatment by topical tacrolimus and/or corticosteroids. A clinical trial is underway to explore predictive biomarkers for dupilumab that are most strongly associated with improvement in EASI.^9^ However, those studies did not investigate whether the biomarkers can predict treatment outcomes. Instead, they investigated how much the biomarkers were associated with treatment outcomes, but an association does not imply prediction since associations often do not generalise to unseen data.^10^ Predictions need to be generated and evaluated on out‐of‐sample data, beyond quantification of associations.

In this study, we explored predictive biomarkers for systemic immunosuppressive therapy for AD (by methotrexate or azathioprine) using the same data as in Roekevisch et al. (2020)^7^ and investigated whether serum cytokines/chemokines measured for each patient pre‐treatment can be used as predictive biomarkers. Here, biomarkers are considered predictive only if their inclusion improves the performance of the best available predictive model (without those biomarkers) for AD severity scores (the primary outcomes of clinical trials). We considered multiple biomarkers in a multivariable regression setting. Comparison with the best available predictive model offsets the effects of other factors, such as historical data, that can help the prediction of future AD severity scores.^11^

Specifically, we developed a statistical machine learning model that can predict the patient‐dependent dynamic evolution of AD severity scores. Our model predicts continuous AD severity scores rather than arbitrary dichotomies of ‘responders’ versus ‘non‐responders’ to avoid potential information loss that may demand us to use more data to reach a reliable conclusion.^12^ Using the model, we explored predictive biomarkers that can reliably predict AD severity scores at different time‐points, not only at a single time point after treatment, to reduce the impact of the variability in treatment responses at an individual patient‐level. A mere comparison of AD severity scores before and after treatment is not suitable to determine patient‐level treatment responses and whether biomarkers are predictive of those responses, because AD severity scores dynamically fluctuate over time regardless of treatment or biomarkers.^12^ Such fluctuations can be stochastic (unpredictable), due to unobserved/unrecorded factors (e.g., environmental factors) or measurement error (cf. inter‐ and intra‐rater variability of severity scores).

## 3 METHODS

### 3.1 Data

We used longitudinal data from a published clinical study^7^ where 42 adult AD patients received systemic therapy (azathioprine or methotrexate) for over 24 weeks. The data includes the baseline concentrations of 26 serum cytokines and chemokines (listed in Figure 4) measured before the start of the treatment (week 0), the status of the filaggrin gene (FLG) mutation (yes/no), age and sex for each of the 42 patients. Therapeutic responses were assessed by EASI, SCORAD, oSCORAD (the objective component of SCORAD) and Patient Oriented Eczema Measure (POEM) at weeks 0, 2, 4, 8, 12 and 24 from the start of the therapy for each patient.

Concentrations of the serum biomarkers were log‐transformed and standardised to have a mean 0 and a variance 1 for each biomarker. Three out of 1092 (= 26 × 42) measurements of the serum biomarkers were missing and imputed by the population mean of the corresponding biomarker. The missing FLG mutation status for six patients was imputed by a default status of ‘no mutation’. The patients' age was standardised to have a population mean of 0 and variance of 1. Our statistical machine learning model (detailed below) considers the dynamics of the severity scores with a constant interval of 2 weeks up to week 24. We therefore treated the absence of the AD severity measurement at weeks 6, 10, 14, 16, 18, 20 and 22 as missing. It resulted in 56% missing values for EASI, (o)SCORAD and POEM.

### 3.2 Model overview

**FIGURE 1:**
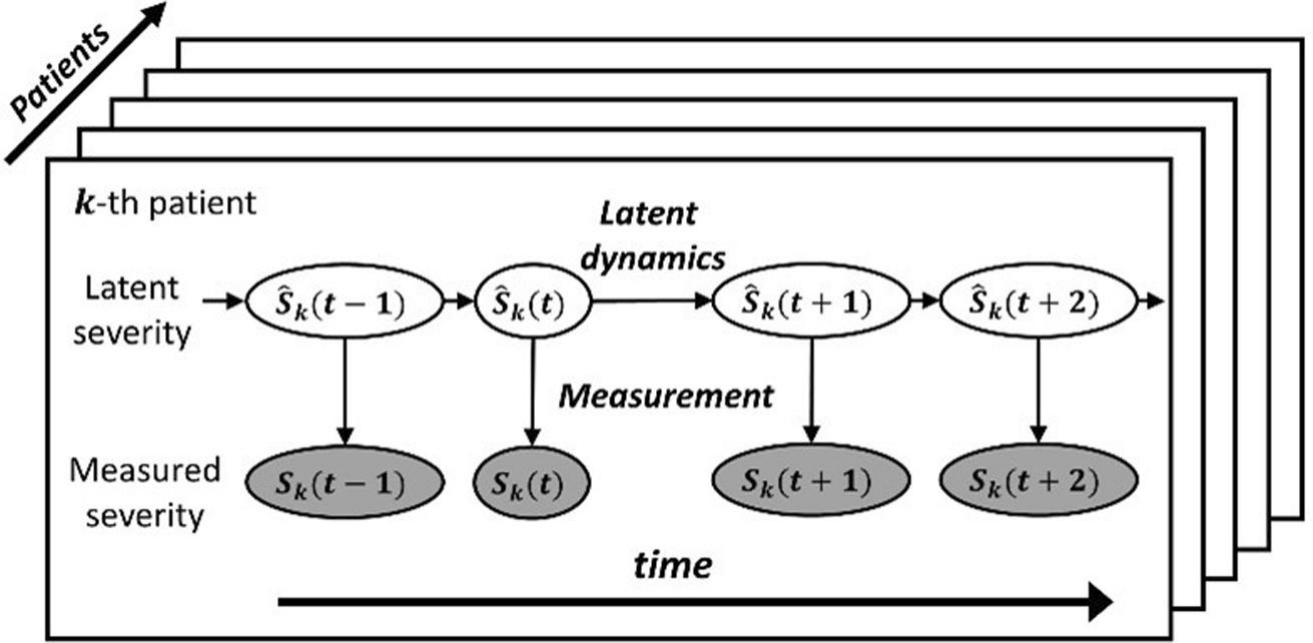
An overview of the Bayesian state‐space model (SSM) for probabilistic predictions of atopic dermatitis (AD) severity scores. The model describes the latent dynamics of a latent severity score (white ovals) and the measurement of the latent severity scores (grey ovals)

We developed a Bayesian state‐space model (SSM) (a statistical machine learning model) to make probabilistic predictions of future AD severity scores (either EASI, SCORAD, oSCORAD or POEM) for each patient. The model for each severity score assumes that the true latent (unobserved) severity score follows its own latent dynamics and that the measured severity score is obtained as a result of an imperfect measurement of the latent severity score at each timepoint (Figure 1). Missing values were treated in our model as an absence of measurement. As a Bayesian model, our model described uncertainties in parameters and severity scores as probability distributions. Quantifying uncertainties in parameters is especially suitable when dealing with small datasets, where the estimates are likely to be noisy.

We modelled the latent dynamics of the latent score, ${\hat{S}}_{k}\left(t\right)$, for the k‐th patient at the t‐th timepoint (with a constant interval of 2 weeks) by a mixed effect autoregressive model, ${\hat{S}}_{k}\left(t+1\right)∼\;N\left(\alpha_{k}{\hat{S}}_{k}\left(t\right)+b_{k}+x_{k}^{T}\beta ,\;\sigma_{l}^{2}\right)$, where $\alpha_{k}$ is the autocorrelation parameter, $b_{k}$ is the intercept, $x_{k}\;$ is an optional covariates vector for the k‐th patient (including biomarkers) with their coefficients, β, and $\sigma_{l}$ is the standard deviation of the latent dynamics. We performed feature selection on the covariates $x_{k}$ by assuming a regularised horseshoe prior for β.^13^ The horseshoe prior shrinks small coefficients toward 0 while allowing strong signals to remain large, thus limiting overshrinkage unlike $L_{1}$ or $L_{2}$ regularisations.^14^

Measurement of the latent score, ${\hat{S}}_{k}\left(t\right)$, is modelled by a truncated Gaussian distribution, $S_{k}\left(t\right)∼N_{\left[0,\;M\right]}\left({\hat{S}}_{k}\left(t\right),\;\sigma_{m}^{2}\right)$, centred around ${\hat{S}}_{k}\left(t\right)$, where $S_{k}\left(t\right)\;$ is the measured severity score for the k‐th patient at the t‐th timepoint. The distribution is truncated between 0 and the maximum value, M, of the severity score (72 for EASI, 83 for oSCORAD, 103 for SCORAD and 28 for POEM). The standard deviation of the measurement process, $\sigma_{m},$ quantifies the measurement error.

We assumed a hierarchical prior for $\alpha_{k}$ and $b_{k}$ and weakly informative priors for the other parameters (detailed in Supplementary A). Model inference was performed using the Hamiltonian Monte‐Carlo algorithm in the probabilistic programming language Stan^15^ with four chains and 2000 iterations per chain including 50% burn‐in. Prior predictive checks and fake data checks were conducted. Convergence and sampling were monitored by looking at trace plots, checking the Gelman‐Rubin convergence diagnostic ($\hat{R})$, and computing effective sample sizes (*N*_eff_).

### 3.3 Model validation

The predictive performance of our model was assessed by *K*‐fold cross‐validation (K=7, stratified by patients) where we applied forward chaining to the ‘test’ fold to reflect how the model would be used in a clinical setting with the model being updated after each measurement (Figure S1). The probabilistic predictions of AD severity scores were evaluated by a logarithmic scoring rule, the log predictive density (lpd), and compared to that of four reference models (detailed in Supplementary B): a uniform forecast model, a random walk model, an autoregressive model and a mixed effect autoregressive model. We also report the root mean squared error of the mean prediction for ease of interpretation.

## 4 RESULTS

### 4.1 Model fit and validation

**FIGURE 2:**
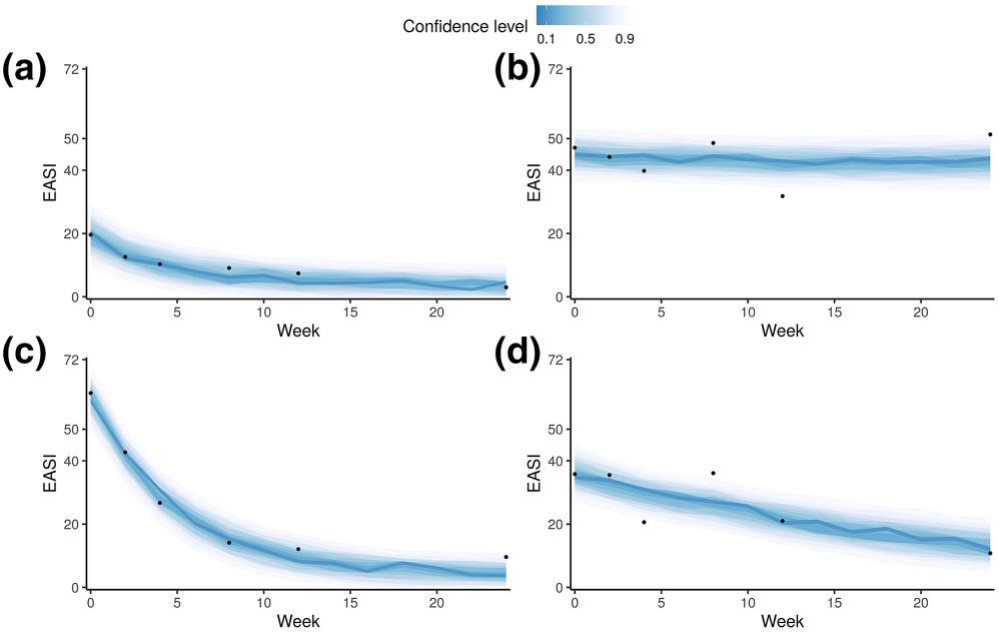
The posterior predictive distribution of four representative patients (a‐d) by our model predicting Eczema Area and Severity Index (EASI) dynamics. Each of the representative patients demonstrates different dynamics: slow recovery from a moderate EASI (a), persistence of severe EASI (b), rapid recovery from a severe EASI (c), and slow recovery from a severe EASI (d). Dots indicate the measured EASI scores, and the coloured ribbons represent stacked credible intervals. Lighter and darker ribbons correspond to wider and narrower highest density credible intervals, respectively

We first developed a Bayesian SSM that predicts the dynamic evolution of AD severity scores without covariates (i.e., without demographics, types of treatment, cytokines/chemokines) as a baseline model. The baseline model that predicts future EASI was fitted successfully to the data without evidence of an absence of convergence (Table S1). Population‐level parameters were estimated with good precision with posterior distributions narrower than their prior distributions (Table S1). We confirmed that the patient‐dependent parameters, $\alpha_{k}$ and $b_{k},$ vary between patients, within the range of [0.37, 0.99] for the expected autocorrelation ($\alpha_{k}$) and [0.03, 2.3] for the expected intercept ($b_{k}$). The measurement process is responsible for 94.7% (90% credible interval 87.3%–99.1%) of the total variance for prediction. The posterior predictive distribution of EASI trajectories demonstrated that the model could capture different patterns, despite the absence of several measurements (Figure 2).

**FIGURE 3:**
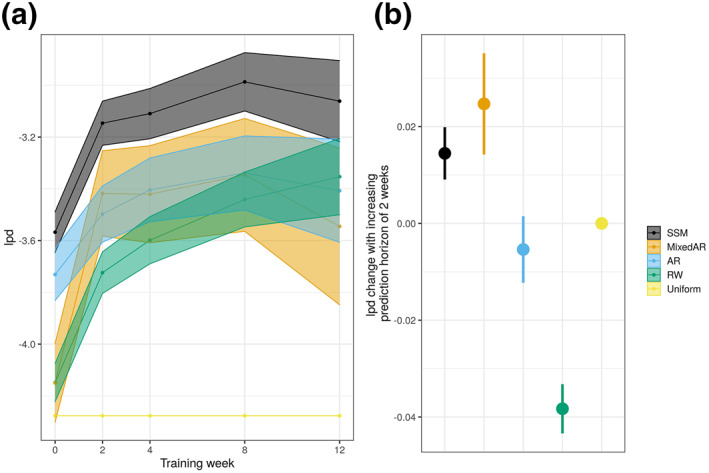
Predictive performance for Eczema Area and Severity Index (EASI) by our Bayesian state‐space model (SSM, black) and the reference models. The performance was evaluated by lpd (higher the better). (a) Learning curves (mean ± SE) for 2‐weeks ahead prediction after adjusting for different prediction horizons. (b) Changes in lpd as the prediction horizon is increased by 2 weeks. The reference models include a mixed effect autoregressive model (MixedAR, orange), an autoregressive model (AR, blue), a random walk model (RW, green), and a uniform forecast model (Uniform, yellow)

Learning curves for two‐weeks ahead predictions of EASI by our Bayesian state‐space model (SSM in Figures 3a and S2) demonstrated that the predictive performance improved as more training data (newer measurements for the same patient) came in and that our model outperformed all the reference models, supporting the structure of our model. The root mean squared error of the mean prediction for EASI at the next clinical visit (e.g., from week 0 to 2, 2 to 4, 4 to 8, etc.) was 6.3±0.62 (mean ± SE) for our model, smaller than 9.9±0.43 for the random walk model. The performance of our model and the mixed autoregressive model for EASI prediction tended to improve as the prediction horizon increased (Figures 3b and S3), while we normally expect the predictive performance decreases for a longer prediction horizon. This counterintuitive observation is possibly because most patients tended to recover before the end of the study, making predictions easier.

Similar results were observed for oSCORAD, SCORAD and POEM by our model, with more measurement error for POEM compared to EASI and (o)SCORAD (Figure S3 and Table S2).

### 4.2 Effects of biomarkers on the model's predictions

**FIGURE 4:**
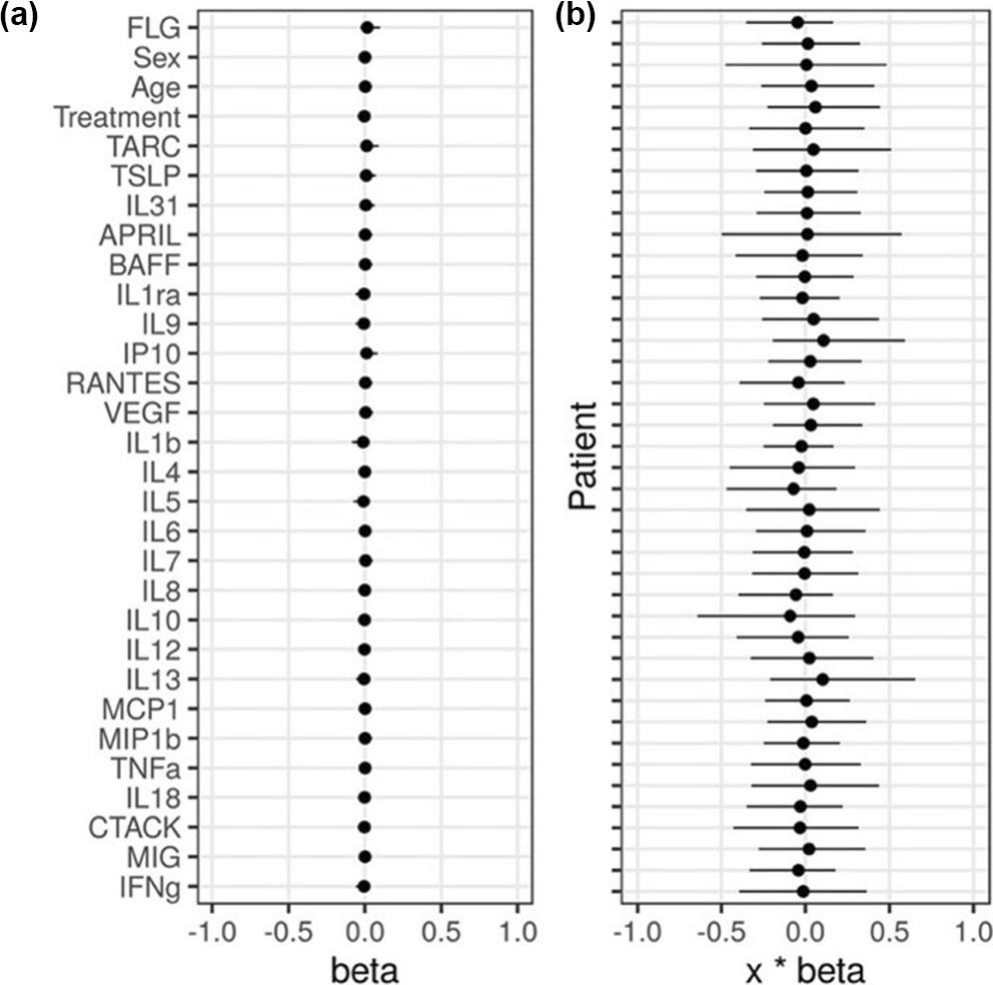
Effects of covariates in our model's predictions of Eczema Area and Severity Index (EASI) (mean and 90% credible intervals). (a) Estimates of the coefficients for the biomarkers (26 serum cytokines/chemokines, filaggrin gene, sex, age) and the treatment applied. A change of one standard deviation in a covariate corresponds to a change of 1.0 in EASI score. (b) Total contribution of all covariates ($x_{k}^{T}\beta$) to EASI prediction for each patient

As our Bayesian SSM outperformed the reference models, we used it to evaluate whether the inclusion of biomarkers improves its predictive performance, thus identifying predictive biomarkers. The covariates included were the 26 serum cytokines/chemokines measured at week 0, the status of FLG mutation, the type of systemic therapy applied (azathioprine or methotrexate), sex and age. Our analysis demonstrated that none of the covariates had a practically significant effect on the model's prediction, as indicated by a small magnitude of the posterior mean and 90% credible intervals for the coefficients, β, on both sides of 0 (Figure 4a), and a resulting small and not practically significant contribution of the covariates ($x_{k}^{T}\beta )$ to the EASI prediction (Figure 4b). As a result, the predictive performance of the model was not improved by including covariates. Similarly, we found no practically significant covariates for the predictive models of SCORAD, oSCORAD and POEM.

## 5 DISCUSSION

Prediction of whether a patient is likely to respond to a specific therapy is of high clinical importance especially if the therapy may have risks of side effects. In this study, we examined whether serum cytokines/chemokines measured for each patient before the start of the therapy can be used as predictive biomarkers for systemic immunosuppressive therapy (methotrexate or azathioprine) for AD.

We developed a Bayesian SSM that can predict AD severity scores (EASI, SCORAD, oSCORAD and POEM) two‐weeks in the future at the individual level. The model describes the dynamics of the latent severity for each patient and the measurement process of the severity scores (Figure 1). The model was trained on the data from 42 adult AD patients who received systemic immunosuppressive therapy in a published clinical study^7^ (Figure 2). Our model outperformed reference models for time‐series forecasting (Figure 3) and was used for further analysis to test the predictive ability of potential predictive biomarkers. The results revealed that the predictive performance was not improved by including some biomarkers as covariates (Figure 4), suggesting that the biomarkers measured before the start of the therapy did not carry information for the prediction of future AD severity scores.

While an absence of evidence for predictive biomarkers of the therapies should not be interpreted as evidence of an absence, our results suggest that the effect of biomarkers on the prediction of severity scores, if any, is likely to be small or too subtle to be captured by our linear model, because the prediction errors of future scores by our model was mostly attributed to errors in the score measurement process. Further investigation of the effect of biomarkers on severity score prediction may therefore require the data from a larger cohort. It is unclear how much new information we can expect to obtain by the inclusion of more biomarkers, because the biomarkers included in this study have been claimed to be most related to AD^4^ and biomarkers are often highly correlated with each other. In addition, the biomarkers' concentrations measured at a single time point are likely to be noisy and may not capture the dynamic heterogeneity of complex diseases such as AD. Whether the benefit of potentially more accurate predictions with biomarkers outweighs the cost of collecting data for such models remains an open question.

While the data used in this study is from a small cohort of patients (*n* = 42), the AD severity scores were measured at six timepoints for each patient. The repeated measurements of severity scores enabled us to capture the dynamic nature of the AD severity scores for each patient and to investigate consistent effects of biomarkers and treatments on AD severity scores within each patient, as it reduces the impact of the variability in treatment responses (including measurement errors).

The analysis of the data in this study did not identify any predictive biomarkers for systemic immunosuppressive therapy for AD, and validation on different cohorts of patients is still required. The method proposed in this study may help to re‐analyse previously collected individual longitudinal data to test the predictive ability of potential predictive biomarkers.

## CONFLICTS OF INTEREST

The authors declare no conflict of interest.

## AUTHOR CONTRIBUTIONS

**G. Hurault:** Conceptualization; Data curation; Formal analysis; Methodology; Writing – original draft. **E. Roekevisch:** Resources. **M. E. Schram:** Resources. **K. Szegedi:** Resources. **S. Kezic:** Resources; Writing – review & editing. **M. A. Middelkamp‐Hup:** Resources; Writing – review & editing. **P. I. Spuls:** Resources; Writing – review & editing. **R. J. Tanaka:** Conceptualization; Funding acquisition; Project administration; Resources; Supervision; Writing – original draft; Writing – review & editing.

## Supporting information

Supporting Information S1

## Data Availability

All the codes used in this study are available at https://github.com/Tanaka‐Group/ssm‐eczema‐biomarkers.
