# Supplementary material for: Can serum biomarkers predict the outcome of systemic immunosuppressive therapy in adult atopic dermatitis patients?
Source: Skin Health Dis. 2022 Jan 7;2(1):e77. doi: 10.1002/ski2.77 (PMC9060148; doi:10.1002/ski2.77)
Supplement: Supplementary file 1 — Supporting Information S1 [file SKI2-2-e77-s001.pdf]

Can serum biomarkers predict the outcome of systemic immunosuppressive therapy in adult atopic dermatitis patients?

SUPPORTING INFORMATION

Guillem Hurault, Evelien Roekevisch, Mandy E. Schram, Krisztina Szegedi, Sanja Kezic, Maritza A. Middelkamp-Hup, Phyllis I. Spuls and Reiko J. Tanaka

A. Priors for Bayesian state-space model (SSM)

We chose the priors for our SSM to be weakly informative. Weakly informative priors are priors designed to rule out unreasonable parameter values (e.g. noise parameters outside the range of the scores) without excluding any values that could make sense. The influence of weakly informative priors is expected to disappear with enough data. We confirmed that our priors were reasonable by conducting prior predictive checks and that our results were not sensitive to the choice of realistic priors.

Instead of defining priors for σ_{m} and σ_{l}, we parametrised the model with $\sigma_t = \sqrt{\sigma_{\mathrm{m}}^2 + \sigma_{\mathrm{l}}^2}$, the standard deviation for two-weeks ahead prediction and $\rho^2 = \frac{\sigma_{\mathrm{m}}^2}{\sigma_{\mathrm{t}}^2}$, the ratio of the measurement variance on the total variance. ρ^2 can be interpreted similarly to an R-squared, the proportion of the explained variance (the variance of the measurements) in the total variance. The priors for σ_{t} and ρ^2 are given by

- $\frac{\sigma_{\mathrm{t}}}{M} \sim \log N(-\log 20, (0.5 \log 5)^2)$, a lognormal prior with a 95% confidence interval of $[0.01M, 0.25M]$, and
- $\rho^2 \sim Beta(4, 2)$, a Beta distribution to reflect our expectation that future severity scores are predictable ($\sigma_{\mathrm{l}} < \sigma_{\mathrm{m}}$).

We assumed a hierarchical prior for the autocorrelation parameter, $\alpha_k \sim \mathrm{Beta}(\mu_\alpha \phi_\alpha, (1-\mu_\alpha)\phi_\alpha)$, where μ_α is the population mean of the Beta distribution and ϕ_α is the population pseudo sample size of the Beta distribution. The priors for μ_α and ϕ_α are given by

- $\mu_\alpha \sim \mathrm{Beta}(2, 2)$, a symmetric Beta distribution that slightly favours values around 0.5 as opposed to 0 or 1, and
- $\phi_\alpha \sim \log N(\log 10,\ (0.5 \log 10)^2)$, a log-normal prior with a 95% confidence interval being approximately $[1, 100]$, allowing a wide variety of distributions for α_k from well spread to concentrated.

We defined the prior for the intercept, b_k, by introducing the expected value of the autoregressive process, S_k^∞, such that $b_k = (1-\alpha_k)S_k^\infty$. We assumed a Gaussian hierarchical prior on $S_k^\infty \sim N(\mu_\infty, \sigma_\infty^2)$, where μ_∞ is the population mean of S_k^∞ and σ_∞ is the population standard deviation of S_k^∞. The priors for μ_∞ and σ_∞ are given by

- $\frac{\mu_\infty}{M} \sim N(0.5, 0.25^2)$, a Gaussian distribution that covers the range $[0, M]$ of the score, and
- $\frac{\sigma_\infty}{M} \sim N^+(0, 0.125^2)$, a half-Gaussian distribution to reflect an assumption that σ_∞ is at most $0.25\,M$, resulting in the width of the distribution for S_k^∞ to be at most M.

We assumed a regularised horseshoe prior for the coefficients, β_i (i=1, …, 30 = D), defined by

- $\beta_i \sim N(0, \tilde{\lambda}_i^2 \tau^2)$, where $\tilde{\lambda}_i = \frac{c^2 \lambda_i^2}{c^2 + \tau^2 \lambda_i^2}$ is the local shrinkage parameter, τ is the global shrinkage parameter and c is the scale of the signal,
- $\lambda_i \sim C^+(0,1)$, where C^+ denotes a half-Cauchy distribution,
- $\tau \sim C^+\left(0, \frac{p_0}{D-p_0} \frac{\sigma}{\sqrt{N}}\right)$, where $p_0 = 5$ is the expected number of covariates with non-zero coefficients, $D = 30$ is the number of covariates, $N = 42$ is the number of patients, and $\sigma = \sigma_{\text{l}}$ is the standard deviation of the residuals, and
- $c^2 \sim$ Inv-$\chi^2(\nu, \sigma_\chi^2)$, a scaled-inverse chi-squared prior, where we assume the degree of freedom, $\nu = 5$, and the scale, $\sigma_\chi = 1$. This prior corresponds to c following a Student-t slab with ν degrees of freedom and scale σ_χ, if β_i is far from 0. This prior reflects an assumption that the order of magnitude of non-zero coefficients is around 1 but could be higher.

To avoid the situation where most of the mass of the non-truncated distribution of the latent score, $\hat{S}_k(t)$, is outside of the range $[0, M]$, which can cause computational problems for the (truncated) measurement distribution, we implemented a soft-uniform prior on $\hat{S}_k(t)$. The soft-uniform prior is defined by the probability density function, $f(x) = \frac{logit^{-1}(x+0.01M) - logit^{-1}(x-1.01M)}{1.02M}$, resulting in an approximately constant density between 0 and *M* (i.e. not prioritising any values in this range) with a slow convergence to 0 (i.e. penalising values) outside this range.

B. Reference models

We implemented four reference models, a uniform forecast model and three models of increasing complexity leading to our state-space model. The models were implemented in a Bayesian setting and provided probabilistic predictions for a fair comparison. These models are more advanced than standard off-the-shelf implementation as missing values are treated as parameters to be inferred in a semi-supervised setting.

- The uniform forecast model (Uniform) is described by $S_k(t) \sim U(0, M)$, where each outcome is assigned the same probability density.
- The random walk model (RW) provides a flat forecast, $S_k(t+1) \sim N(S_k(t), \sigma^2)$, centred on the last observation with the uncertainty quantified by a variance, σ^2. The prior for σ is the same as that for σ_{t} in our SSM.
- The autoregressive model (AR) is an extension of the random walk model and is described by $S_k(t+1) \sim N(\alpha\, S_k(t) + b, \sigma^2)$, with a fixed autocorrelation, α, and an intercept, $b = (1-\alpha) S^\infty$. We assumed a uniform prior for $\alpha \sim U(0,1)$. The prior for S^∞ is the same as that for μ_∞ in our SSM.
- The mixed effect autoregressive model (MixedAR) extends the autoregressive model and is described by $S_k(t+1) \sim N(\alpha_k\, S_k(t) + b_k, \sigma^2)$, with a patient-dependent autocorrelation, α_k, and a patient-dependent intercept, $b_k = (1-\alpha_k) S_k^\infty$. The priors for α_k and b_k are the same as those in our SSM.

Table S1: Posterior summary statistics of the population-level parameters for the model predicting EASI without covariates.

Parameter	Interpretation	$\hat{R}$	N_{eff}	Mean	SD	2.5%	50%	97.5%	PS*
σ_t	Square root of the total variance	1.000	1760	4.499	0.283	3.976	4.488	5.094	0.988
ρ^2	Proportion of the σ_{m}^2 in the total variance	1.002	1055	0.947	0.037	0.853	0.955	0.993	0.955
σ_{l}	Standard deviation of the latent dynamic	1.003	1089	0.968	0.340	0.371	0.946	1.671	0.939
σ_{m}	Standard deviation of the measurement process	1.001	1453	4.379	0.310	3.794	4.372	5.013	0.982
MDC	Minimum Detectable Change for a 95% confidence level	1.001	1453	8.582	0.607	7.436	8.568	9.826	0.982
μ_α	Population mean of the autocorrelation parameter α	1.002	1572	0.688	0.042	0.600	0.691	0.765	0.961
ϕ_α	Population "pseudo sample size" of the autocorrelation α	1.004	1174	3.664	1.094	2.026	3.504	6.187	0.999
μ_∞	Population mean of the autoregressive process	1.002	1597	3.574	0.588	2.446	3.561	4.789	0.997
σ_∞	Population standard deviation of the autoregressive process	1.002	2017	0.507	0.405	0.017	0.421	1.489	0.986

* The Posterior Shrinkage (PS) of the parameter θ is defined as $1 - \frac{\text{Var}(\theta_{\text{post}})}{\text{Var}(\theta_{\text{prior}})}$. PS near 0 indicates that the data provides little information beyond the prior and PS near 1 indicates that the data is much more informative than the prior.

Table S2: Measurement error comparison for different severity scores.

	EASI	SCORAD	oSCORAD	POEM
**σ_{m} (mean and 90% credible interval)**	4.38 [3.88, 4.90]	5.79 [4.71, 6.85]	4.65 [3.76, 5.46]	3.94 [3.40, 4.50]
**Mean(σ_{m}) / Range of the scores (%)**	4.38/72 = 6.1%	5.79/103 = 5.6%	4.65/83 = 5.6%	3.94/28 = 14.1%
**ρ^2 (mean and 90% credible interval)**	0.947 [0.873, 0.991]	0.688 [0.471, 0.891]	0.710 [0.482, 0.911	0.886 [0.755, 0.977]

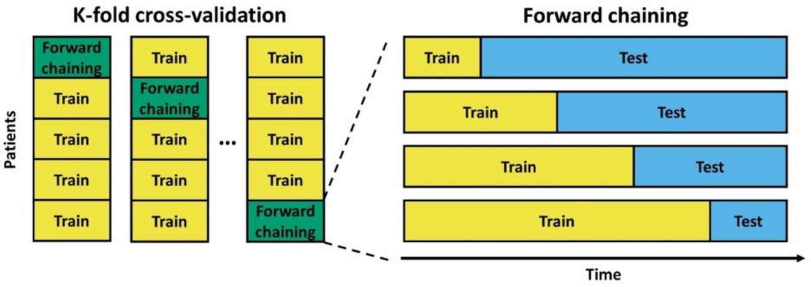

Figure S1: K-fold cross-validation ($K = 5$ in this example) in a forward chaining setting, which reflects how the model would be used in a clinical setting. For each fold, the model was pre-trained with ($K - 1$) subsets of patients and validated on the remaining subset of patients in a forward chaining setting, in which the model was trained with the first timepoint and tested on the remaining timepoints, then the model was trained with the first two timepoints and tested on the remaining timepoints, etc.

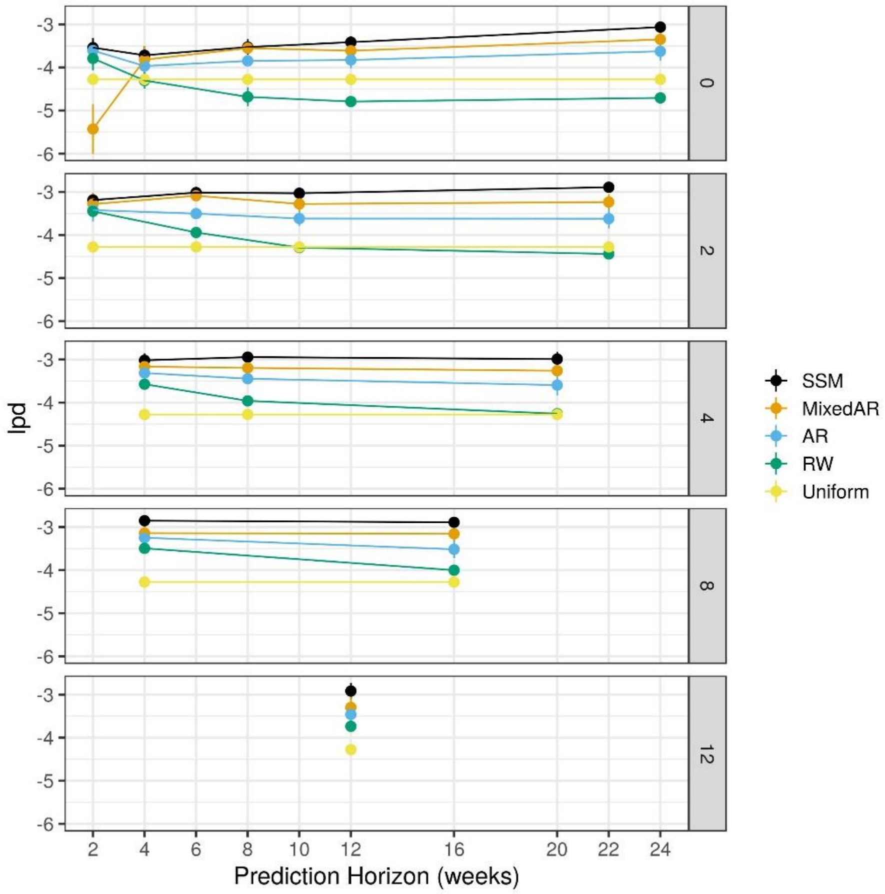

Figure S2: Performance of our model (SSM) and reference models (MixedAR, AR, RW and Uniform) to predict EASI. The performance was evaluated by lpd (higher the better). The values of lpd as a function of the prediction horizon for various training weeks (panels) and models (colours).

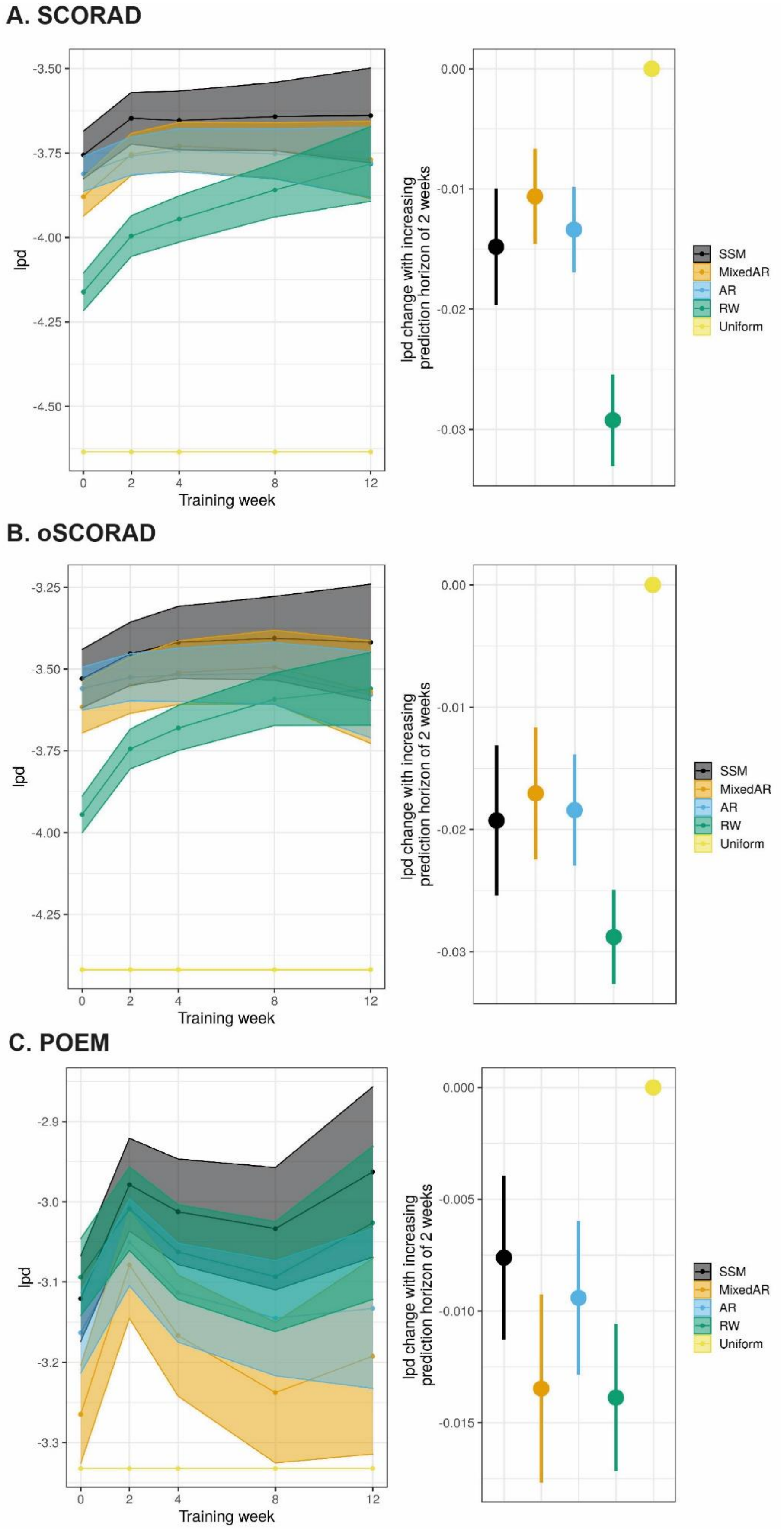

Figure S3: Predictive performance of our model (SSM) and reference models (MixedAR, AR, RW and Uniform) for oSCORAD (**A**), SCORAD (**B**) and POEM (**C**). The performance was evaluated by lpd (higher the better). Left: Learning curves (mean ± SE) for two-weeks ahead prediction after adjusting for different prediction horizons. Right: Change in lpd as the prediction horizon is increased by two weeks.
